# Genetic Investigation of Corrected QT Interval Sensitivity to Oral Bepridil Hydrochloride Hydrate in Patients With Atrial Fibrillation

**DOI:** 10.1161/JAHA.125.047046

**Published:** 2026-06-09

**Authors:** Naoki Ishibashi, Motoki Furutani, Mika Nakashima, Shunsuke Ishida, Junji Maeda, Takumi Sakai, Naoto Oguri, Shogo Miyamoto, Shunsuke Miyauchi, Sho Okamura, Yousaku Okubo, Takehito Tokuyama, Noboru Oda, Takumi Nishiki, Risa Mitsumori, Shumpei Niida, Kouichi Ozaki, Daichi Shigemizu, Hideki Itoh, Yukiko Nakano

**Affiliations:** ^1^ Department of Cardiovascular Medicine Hiroshima University Graduate School of Biomedical and Health Sciences Hiroshima Japan; ^2^ Medical Genome Center Research Institute, National Center for Geriatrics and Gerontology Aichi Japan; ^3^ Research Institute, National Center for Geriatrics and Gerontology Aichi Japan; ^4^ RIKEN Center for Integrative Medical Sciences Yokohama Japan; ^5^ Division of Patient Safety Hiroshima University Hospital Hiroshima Japan

**Keywords:** bepridil, genome‐wide association study, QTc prolongation, Atrial Fibrillation, Biomarkers, Genetics

## Abstract

**Background:**

Bepridil is an antiarrhythmic drug used to treat paroxysmal atrial fibrillation; however, its use is limited by corrected QT interval (QTc) prolongation and the risk of ventricular arrhythmias. This study aimed to identify genetic variants associated with bepridil‐associated QTc prolongation after catheter ablation for paroxysmal atrial fibrillation.

**Methods:**

A total of 523 patients who initiated bepridil therapy after catheter ablation at Hiroshima University between November 2013 and March 2023 were enrolled. Of these, 445 patients were included in a genome‐wide association study, and 78 were used as an independent replication cohort. QTc was measured 1 month after treatment initiation, with QTc prolongation defined as ≥450 ms in men and ≥460 ms in women. Logistic regression analyses were conducted using additive, dominant, and recessive genetic models. Variants showing suggestive associations in the genome‐wide association study (*P*<1.00×10^−6^) were evaluated in the replication cohort, followed by random‐effects meta‐analyses, with genome‐wide significance defined as *P*<5.00×10^−8^. Linear regression analyses were conducted for QTc prolongation.

**Results:**

After quality control, 443 patients were analyzed in the genome‐wide association study. Of these, 122 (28%) exhibited QTc prolongation. Under the recessive model, rs12622919, an intronic variant in the *FSHR* (follicle‐stimulating hormone receptor) gene, showed a suggestive association. In the random‐effects meta‐analysis combining the discovery and replication cohorts, rs12622919 reached genome‐wide significance. Exploratory sex‐stratified analyses suggested a potential association in women (*P*=0.026), although no statistically significant genotype‐by‐sex interaction was observed. Inclusion of rs12622919 improved discrimination for QTc prolongation (area under the curve, 0.77 [95% CI, 0.70–0.84]; Welch's *t* test, *P*<0.001), with the improvement remaining significant by DeLong's test (area under the curve, 0.78 [95% CI, 0.74–0.84]; *P*=0.0078).

**Conclusion:**

The *FSHR* variant rs12622919 is associated with bepridil‐associated QTc prolongation and may serve as a genetic biomarker for individual risk assessment. Because the findings are based on meta‐analysis, independent validation is required.

**Nonstandard Abbreviations and Acronyms**
eQTL: expression quantitative trait loci
FSH: follicle‐stimulating hormone
FSHR: follicle‐stimulating hormone receptor
GTEx: Genotype‐Tissue Expression
IKr: rapid delayed rectifying potassium current
PAF: paroxysmal atrial fibrillation
PPP1R21: protein phosphatase 1 regulatory subunit 21
QTc: corrected QT interval
RFCA: radiofrequency catheter ablation

**Clinical Perspective**

**What Is New?**

- This genome‐wide association study identified rs12622919 in the *FSHR* gene as a genetic variant associated with bepridil‐associated corrected QT interval prolongation.

**What Are the Clinical Implications?**

- Genetic variation in rs12622919 may be associated with susceptibility to bepridil‐associated corrected QT interval prolongation; with exploratory sex‐stratified analyses suggesting a potential effect in women.
- Incorporating rs12622919 into a predictive model enhanced the risk stratification for corrected QT interval prolongation, suggesting its potential usefulness as a biomarker for a safer and more personalized antiarrhythmic therapy.

Acquired long QT syndrome is characterized by a prolonged QT interval and the development of potentially life‐threatening ventricular arrhythmias triggered by external factors, such as medications (eg, antiarrhythmic agents, antibiotics), electrolyte imbalances, and bradycardia.^1^ In congenital long QT syndrome, genetic mutations underlie the disease mechanism, with loss‐of‐function mutations in potassium channel genes and gain‐of‐function mutations in sodium or calcium channel genes, resulting in delayed cardiac repolarization and the development of malignant ventricular arrhythmias.^1^, ^2^ Furthermore, recent studies have shown that genetic predisposition is involved not only in congenital long QT syndrome but also in acquired long QT syndrome, and mutations in genes associated with congenital long QT syndrome have been identified in patients with acquired long QT syndrome, emphasizing the importance of genetic background even in acquired diseases.^3^ This growing recognition has driven interest in the genetic investigation of acquired long QT syndrome to better understand individual susceptibility and risk stratification. A multicenter study from Japan and Europe revealed that patients with drug‐induced torsades de pointes could carry a variant in the major congenital long QT syndrome candidate genes (*KCNQ1*, *KCNH2*, *SCN5A*, *KCNE1*, and *KCNE2*),^4^ and the international guidelines^5^ have recommended genetic testing for patients aged <40 years with drug‐induced torsades de pointes.^5^ In addition, the common genetic variants influencing corrected QT interval (QTc) prolongation have also been investigated.^6^, ^7^, ^8^, ^9^ These widely distributed genetic findings suggest that the genetic background may be heterogeneous and vary depending on the characteristics of each patient with drug‐induced QTc prolongation.

Bepridil hydrochloride hydrate (Bepricor; Kowa Pharmaceutical Co., Ltd., Japan) is a multi‐ion channel blocker used for the prevention and treatment of paroxysmal atrial fibrillation (PAF).^10^, ^11^, ^12^, ^13^ Despite the clinical efficacy of bepridil, it has been associated with a known risk of QT interval prolongation and fatal ventricular arrhythmias, such as torsades de pointes.^14^, ^15^ Given that the degree of QTc prolongation varies among individuals, it is extremely important to identify patients who are susceptible to bepridil‐induced QTc prolongation.

This study aimed to investigate the genetic background of bepridil‐induced QTc prolongation in patients who underwent catheter ablation for PAF and subsequently received oral bepridil therapy. We focused on common genetic variants, specifically, single nucleotide polymorphisms (SNPs), and conducted a genome‐wide association study (GWAS) to identify the genetic factors contributing to susceptibility to QTc prolongation during bepridil treatment.

Although most GWASs use additive models, recessive models have also been shown to be useful. For example, several variants with recessive effects that could not be detected by additive models have been identified in studies on type 2 diabetes.^16^ In addition, in the context of drug metabolism, *CYP2C19* polymorphisms serve as a representative example, as individuals carrying loss‐of‐function variants in both alleles tend to exhibit significantly reduced enzyme activity and have an increased risk of thrombotic events.^17^

Therefore, we performed not only an additive model analysis but also dominant and recessive model analyses to achieve a comprehensive investigation. Our findings may provide insights into personalized risk prediction and safer antiarrhythmic strategies.

## Methods

### Data Availability

All data sets used or analyzed are available from the corresponding author upon reasonable request.

### Patient Recruitment

This study enrolled a total of 523 patients with PAF who received treatment at the Department of Cardiovascular Medicine, Hiroshima University, between November 2013 and March 2023. All enrolled patients underwent a first‐time radiofrequency catheter ablation (RFCA) and were started on oral bepridil hydrochloride hydrate (Bepricor; Kowa Pharmaceutical Co., Ltd., Nagoya, Japan) at a daily dose of 100 mg on the day after the procedure. Clinical data, including demographic characteristics (age, sex, and body mass index [BMI]), were collected from all patients. QTc was measured at baseline and 1 month after the initiation of bepridil (baseline QTc and posttreatment QTc, respectively). Based on measurements taken 1 month after bepridil administration, the definition of QTc prolongation was established as ≥450 ms in men and ≥460 ms in women on the basis of reliable reports.^18^, ^19^ Patients whose QTc already met or exceeded these thresholds before bepridil administration were excluded. Participants taking other medications with QT‐prolonging effects (eg, antibiotics, antipsychotics, and antidepressants) were considered to be at high risk for arrhythmias associated with QTc prolongation. Therefore, bepridil was not actively prescribed for individuals receiving these drugs in our institute. However, only a small number of patients were already taking these medications (Table 1 and Table S1), and no significant difference was observed in medication use between the QT prolongation group and the non‐QT prolongation group. Additional exclusion criteria were (1) outlier values (defined as ±4 SDs from the mean) for age, QTc, or BMI; (2) ECGs not in sinus rhythm, including atrial or ventricular pacing due to implanted cardiac devices; and (2) a diagnosis of congenital long QT syndrome, family history of sudden cardiac death, or other high‐risk phenotypes.

**Table 1:** Demographic Data of a GWAS Cohort

Clinical information	QTc prolongation* (n=122)	Non–QTc prolongation (n=323)	*P* value
Age, y	66.00±11	63.30±11.3	0.016
Sex, female	39 (31.97)	101 (31.27)	0.91
BMI, kg/m^2^	23.20±3.58	24.10±3.48	0.016
Baseline QTc, ms	429±16.90	416±19.00	5.06E‐12
Posttreatment QTc, ms	465±17.20	431±17.40	3.38E‐55
Heart rate, bpm	62.60±9.05	62.36±9.72	0.65
Blood test			
Aspartate aminotransferase, U/L	23.12±7.07	22.78±7.31	0.48
Alanine aminotransferase, U/L	21.91±11.35	22.30±10.71	0.57
γ‐glutamyltransferase, IU/L	36.64±38.75	36.02±31.78	0.49
Creatinine, mg/dL	0.84±0.18	0.81±0.18	0.60
Estimated glomerular filtration rate, mL/min per 1.73 m^2^	68.98±13.95	70.76±15.49	0.27
Sodium, mEq/L	140.11±1.68	139.92±2.08	0.59
Potassium, mEq/L	4.10±0.28	4.14±0.25	0.051
Chloride, mEq/L	105±2.35	104.90±2.46	0.88
Antiarrhythmic drugs			
Na channel blocker			
Ia	7 (5.74)	43 (13.31)	0.028
Ib	0 (0)	0 (0)	
Ic	13 (10.66)	46 (14.24)	0.35
Amiodarone	6 (4.92)	20 (6.19)	0.82
QTc concomitant drugs			
Antibiotics	1 (0.82)	1 (0.3)	0.47
Antipsychotics	2 (1.6)	3 (0.93)	0.62
Antidepressants	0 (0)	3 (0.93)	0.57
Antihistamines	2 (1.6)	4 (1.24)	0.67
Antiepileptic drugs	1 (0.82)	3 (0.93)	1
Proton pomp inhibitor	68 (55.73)	148 (45.82)	0.071
Magnesium oxide	7 (5.74)	10 (3.10)	0.26
Herbal medicine	7 (5.74)	15 (4.64)	0.63

Values are reported as mean±SD or n (%). BMI indicates body mass index; GWAS, genome‐wide association study; and QTc, corrected QT interval.

* QTc prolongation was defined as a QTc of ≥450 ms in men and ≥ 460 ms in women.

Genomic DNA was extracted from the peripheral blood leukocytes in accordance with the standard protocols using the Maxwell RSC Buffy Coat DNA kit (Promega, Madison, WI). Written informed consent was obtained from all patients. The study protocol was approved by the ethics committees of the National Center for Geriatrics and Gerontology (No. 990‐16) and Hiroshima University (Approval No. E2002‐9952) in accordance with the Declaration of Helsinki. The raw data that support the findings of this study are available from the corresponding author upon reasonable request.

### QTc Measurement

The baseline QTc was defined as the QTc measured on ECGs obtained at hospital admission for RFCA. Posttreatment QTc was defined as the QTc measured on ECGs recorded at the 1‐month outpatient follow‐up after initiation of oral bepridil (100 mg/d). The QTc was measured in lead V5 and corrected with Bazett's formula, manually and independently by at least 2 cardiologists specializing in arrhythmias, and we adopted the measurement only if the difference was <5%. Amiodarone was discontinued at least 2 weeks before RFCA, and other antiarrhythmic drugs were discontinued 1 week before the procedure.

### Genome‐Wide Association Study

Genotyping for the GWAS was conducted using the Asian screening arrays (Illumina, San Diego, CA). Genotype imputation was performed using Minimac4 with a 5.6 K in‐house reference panel from the National Center for Geriatrics and Gerontology biobank.^20^ The imputed variants with an Info score of ≥0.3 were included in the subsequent analysis. The samples that met the following quality control (QC) criteria were used: a sample call rate of ≥99%, genotype missingness of <1%, correct sex specification, absence of close genetic relationships (PI_HAT >0.25 calculated by PLINK software^21^), and exclusion of outliers base on clustering within East Asian populations identified by principal component analysis using the 1000 Genomes Project phase 3 data (https://www.internationalgenome.org). The QC filters were applied to the genetic markers, including a genotyping efficiency call rate of ≥99%, minor allele frequency of >1%, and Hardy–Weinberg equilibrium *P* value >1.00×10^−6^ in controls. Common autosomal variants that met the QC criteria were analyzed using a logistic regression model, with adjustment for age, sex, BMI, and the top 3 principal components, using PLINK software. Three genetic models were used: additive, dominant, and recessive models. The association results were visualized using quantile–quantile and Manhattan plots created with the R package “qqman” (https://github.com/stephenturner/qqman). The lead SNPs identified by GWAS were visualized using LocusZoom (http://locuszoom.org),^22^ which is a tool for visualizing GWAS results with regional plots. The allele frequencies of the variants in the Japanese population were obtained from the Tohoku Medical Megabank Organization database (https://jmorp.megabank.tohoku.ac.jp).^23^

### Replication Study

We identified the variants that met the suggestive *P* value threshold (*P*<1.00×10^−6^) in the GWAS results. These candidate variants were genotyped in 78 patients enrolled between 2022 and 2023, using a multiplex polymerase chain reaction–based Invader assay (Third Wave Technologies, Madison, WI) and QuantStudio 7 Flex Real‐Time Polymerase Chain Reaction System (Thermo Fisher Scientific, Waltham, MA).^24^ Before genotyping, the allele frequencies obtained from the GWAS results were compared with those reported in the Tohoku Medical Megabank Organization database (https://jmorp.megabank.tohoku.ac.jp).^23^ The primers for polymerase chain reactions were designed using the Primer3Plus program (http://primer3plus.com/cgi‐bin/dev/primer3plus.cgi).^25^ All of the primers were synthesized commercially (Fasmac, Kanagawa, Japan). Table S2 shows the primer sequences used for genotyping.

### Meta‐Analysis

After SNP genotyping in the replication cohort, we calculated the odds ratio for each SNP adjusted for age, sex, and BMI. Given the clinical and methodological differences between the discovery GWAS and the independent replication cohort, including differences in sample size, enrollment period, and potential variation in patient characteristics and environmental exposures, we applied a random‐effects meta‐analysis to combine summary statistics from the discovery GWAS and the replication cohort using METASOFT.^26^ Meta‐analysis *P* values were estimated on the basis of Han and Eskin's modified random‐effects model. The significance threshold was set at *P*<5.00×10^−8^.

### Quantitative Trait Locus Analysis of SNPs

The expression quantitative trait loci (eQTL) analyses that were conducted for the lead SNPs identified in the discovery GWAS cohort were examined using the data from the Genotype‐Tissue Expression (GTEx) database version 8 (http://ftp.ebi.ac.uk/pub/databases/spot/eQTL/imported/GTEx_V8/).^27^ The examined tissues included the atrial appendage, left ventricle, kidney, and liver tissues. Variants with a *P* value of <0.05 in genes with normalized transcriptome per million of ≥1 were considered significant.

### Correlation Between the Lead SNP and Functional Coding Variants

To investigate the relationship between the lead SNPs and nearby functional coding variants, the whole‐genome sequencing data from 4344 individuals in the National Center for Geriatrics and Gerontology Biobank were analyzed.^28^ The variant annotation for single nucleotide variants and insertions and deletions located within ±1 Mb of each lead SNP was conducted using ANNOVAR version 2019‐10‐24. The pairwise linkage disequilibrium between the lead SNPs and functional variants was assessed using PLINK version 1.9 with the ‐‐r2 and ‐‐ld‐window options. The Pearson correlation coefficients between the lead SNPs and functional variants were calculated using the R software (R Foundation for Statistical Computing, Vienna, Austria). Statistical significance was defined as a false discovery rate of <0.05.

### Association Analysis Between the Candidate SNP and Clinical Factors

After identifying the candidate SNP through a meta‐analysis, we performed linear regression analyses to evaluate its association with QTc prolongation. Sex‐stratified linear regression analyses were additionally conducted to explore potential sex‐specific associations. Furthermore, a genotype‐by‐sex interaction analysis was performed using PLINK (‐‐interaction) to assess effects by sex.

### Comparing Performance With and Without the Candidate SNP

The data set was randomly split into training (75%) and test (25%) sets. Two logistic regression models were trained: a clinical model including age, sex, BMI, and baseline QTc, and a genetic model that additionally incorporated the candidate SNP. Model performance was evaluated by the area under the curve (AUC) on the test set. To assess the robustness, the procedure was repeated 1000 times with bootstrap resampling, and 95% CIs were estimated. Differences in AUC between the 2 models were compared using DeLong's test.

### Statistical Analysis

For the demographic data of the study participants, significant differences in the categorical variables between the QTc prolongation and non‐QTc prolongation groups were assessed using a Fisher's exact test, whereas significant differences in the continuous variables between the QTc prolongation and non‐QTc prolongation groups were evaluated using the Mann–Whitney *U* test. These analyses were performed using SciPy in the Python package version 1.7.3 (Python Software Foundation, Wilmington, DE).

An association analysis between the candidate SNP and posttreatment QTc was conducted using a linear regression adjusted for age, sex, BMI, and the baseline QTc. Sex‐stratified analyses between the candidate SNP and posttreatment QTc were also performed, adjusting for age, BMI, and baseline QTc. These analyses were performed using the Python package statsmodels version 0.13.2. Predictive model construction, AUC calculation, and bootstrap resampling were implemented using scikit‐learn version 1.4.2. DeLong's test was conducted using the R package pROC version 1.18.15. Differences in predictive performance between models were assessed using Welch's *t* test. A *P* value of <0.05 was considered statistically significant.

## Results

### Demographic Data of the GWAS Participants

Of the 445 patients (women, 31.46%; mean age, 64.04±11.29 years), 122 (women, 31.97%; mean age, 66.00±11.00 years) experienced QTc prolongation, whereas the remaining 323 did not (women, 31.27%; mean age, 63.30±11.30 years). The QTc prolongation group had a higher mean age and lower mean BMI compared with the non‐QTc prolongation group. The baseline QTc and posttreatment QTc were longer in the QTc prolongation group than in the non–QTc prolongation group. Blood tests showed no differences among liver function, kidney function, or electrolytes between the 2 groups. Regarding concomitant medication, the use of class Ia antiarrhythmic drugs was more frequent in the non–QTc prolongation group than in the QTc prolongation group; however, no significant difference was observed in the use of other antiarrhythmic drugs. No significant difference was observed between the 2 groups in the use of other concomitant medications (Table 1).

### GWAS Results

**Figure 1:**
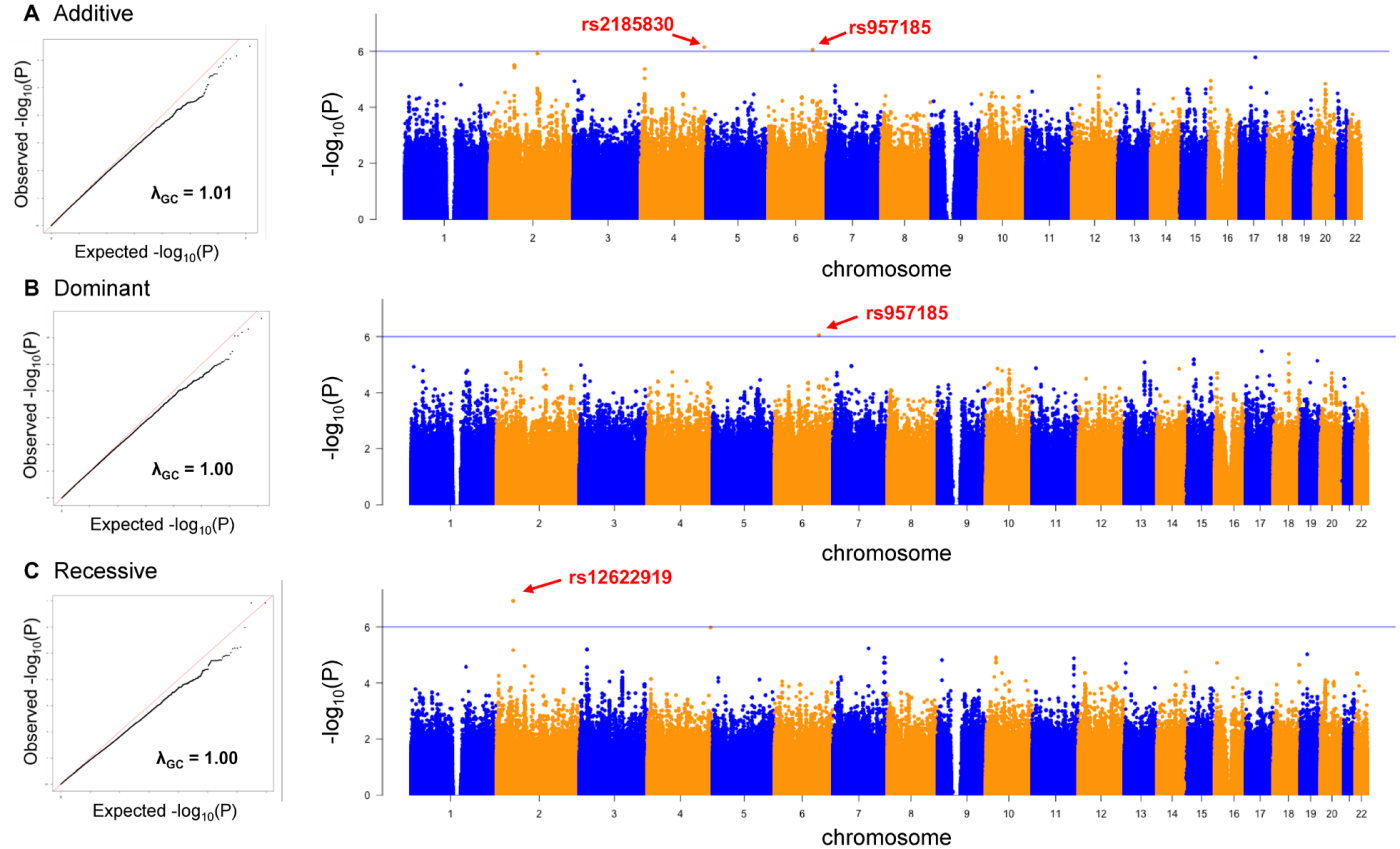
Results of the GWAS additive, dominant, and recessive models. The Manhattan plot of the GWAS results. Suggestive significance was defined using a *P* value of 1×10^−6^, as indicated by the blue horizontal line. The red arrows indicate 4 variants that reached this suggestive significance level in at least one of the (**A**) additive, (**B**) dominant, and (**C**) recessive models. GWAS indicates genome‐wide association study.

A total of 6 981 631 genetic markers from 443 patients, comprising 121 and 322 patients with and without QTc prolongation, respectively, passed the stringent QC criteria for both genotypes and samples (2 patients were excluded during QC [PI_HAT >0.25]). The quantile–quantile plots of the genome‐wide *P* values across the 3 models revealed no genomic inflation (genomic inflation factor, 1.00–1.01; Figure 1). Three variants reached a suggestive significance: rs2185830 in the additive model, rs957185 in both the additive and dominant models, and rs12622919 in the recessive model (Figure 1 and Figure S1). All of the identified variants were associated with an increased risk, with odds ratios ranging from 2.32 to 7.92 in the GWAS results (Table 2).

### Replication Study and Meta‐Analysis

Three suggestive variants were genotyped in an independent replication cohort. Table S1 summarizes the demographic data of the replication cohort. Logistic regression analyses were performed with adjustment for age, sex, and BMI. Of the 3 variants examined, rs12622919 showed a nominal significance (*P*=0.024; Table 2); however, none of the SNPs reached the Bonferroni‐corrected significance threshold of 0.05 of 3 (Table 2). A meta‐analysis combining the summary statistics from the discovery GWAS and the replication cohort for rs12622919 reached a genome‐wide significance (odds ratio, 3.88 [95% CI, 2.44–6.11]; *P*=8.52×10^−9^; Table 2).

**Table 2:** Summary of Genome‐Wide Association Studies

Model	chr	Position (hg19)	A1	A2	rsID	Nearest gene	Variant annotation	Stage (sample size)	Odds ratio (95% CI)	*P* value	A1/A1A2/A2		A1 AF		Meta*	
											Case	Control	GWAS	ToMMo 54KJPN	Odds ratio (95% CI)	*P* value
Additive	4	185 302 070	C	T	rs2185830	*LINC02362*	Intronic variant	GWAS (n=443)	2.32 (0.17–3.23)	7.09E‐07	42/58/21	44/175/103	0.47	0.44	1.58 (0.64–3.91)	0.16
								Replication (n=78)	0.90 (0.38–2.16)	0.82	4/6/7	10/37/14	0.46			
	6	130 287 785	C	T	rs957185	*L3MBTL3*		GWAS (n=443)	7.92 (3.47–18.07)	8.84E‐07	0/21/100	0/10/312	0.035	0.032	2.32 (0.15–36.15)	0.39
								Replication (n=78)	0.47 (0.053–4.12)	0.49	0/1/16	1/6/54	0.058			
Dominant	6	130 287 785	C	T	rs957185	*L3MBTL3*		GWAS (n=443)	7.92 (3.47–18.07)	8.84E‐07	0/21/100	0/10/312	0.035	0.032	2.36 (0.15–36.66)	0.40
								Replication (n=78)	0.47 (0.049–4.44)	0.51	0/1/16	1/6/54	0.058			
Recessive	2	49 306 289	C	T	rs12622919	*FSHR*	Intronic variant	GWAS (n=443)	3.87 (2.34–6.34)	1.18E‐07	45/44/32	46/189/87	0.47	0.45	3.88 (2.44–6.11)	8.52E‐09
								Replication (n=78)	3.98 (1.20–13.18)	0.024	1/6/10	20/24/17	0.46			

AF indicates allele frequency; chr, chromosome; GWAS, genome‐wide association study; and ToMMo, Tohoku Medical Megabank Organization.

* The meta‐*P* values were calculated using random‐effect models implemented in METASOFT.

### eQTL Effect of rs12622919

We examined the eQTL effects of the GWAS signal, rs12622919, in the heart tissues (atrial appendage and left ventricle) using the data from the GTEx database.^27^ The risk allele of rs12622919 was significantly associated with decreased *PPP1R21* (protein phosphatase 1 regulatory subunit 21) expression in the left ventricle (*P*<0.05; Table S3). Given that our study focused on drug‐induced effects, we also evaluated the eQTL effects of rs12622919 in the liver and kidney (organs that are involved in drug metabolism). However, no significant associations were observed in these organs.

### Correlation Between rs12622919 and Functional Variants

Using the whole‐genome sequencing data from 4344 individuals in the National Center for Geriatrics and Gerontology biobank, we searched for functional coding variants located within ±1 Mb of rs12622919 that show a strong linkage disequilibrium (ie, *r* ^2^>0.8) with this SNP. Although a total of 141 functional variants were identified within this region (Table S4), none of the coding variants showed a strong linkage disequilibrium with rs12622919.

### Associations Between rs12622919 and Clinical Factors

A linear regression analysis, including all participants, showed that rs12622919 was significantly associated with QTc prolongation (β=0.25 [95% CI, 0.072–0.43]; *P*<0.05; Table 3). rs12622919 is located in an intronic region of the *FSHR* (follicle‐stimulating hormone receptor) gene, which plays a key role in gonad development. Given this biological relevance, sex‐stratified linear regression analyses were conducted. The association between rs12622919 and QTc prolongation was significant in women only (β=0.39 [95% CI, 0.046–0.74]; *P*<0.05; Table 3). However, no significant genotype‐by‐sex interaction was observed in the GWAS (recessive interaction term, *P*=0.77); therefore, the sex‐specific finding should be considered exploratory.

**Table 3:** Results of the Linear Regression Analyses

Variable	Factor	Coefficient (95% CI)	*P* value
All participants*	Age	0.069 (−0.010 to 0.15)	0.085
	Sex	0.088 (−0.080 to 0.26)	0.30
	BMI	−0.11 (−0.19 to −0.039)	0.030
	Baseline QTc	0.44 (0.37 to 0.52)	3.88E‐40
	rs12622919	0.25 (0.072 to 0.43)	0.0060
Men†	Age	0.071 (−0.010 to 0.15)	0.085
	BMI	−0.092 (−0.18 to −0.0020)	0.044
	Baseline QTc	0.47 (0.37 to 0.56)	1.15E‐23
	rs12622919	0.16 (−0.049 to 0.37)	0.14
Women†	Age	0.066 (−0.014 to 0.27)	0.63
	BMI	−0.15 (−0.29 to 0.050)	0.043
	Baseline QTc	0.41 (0.27 to 0.55)	1.64E‐08
	rs12622919	0.39 (0.046 to 0.74)	0.026

BMI indicates body mass index; and QTc, corrected QT interval.

* Linear regression adjusted for age, sex, BMI, baseline QTc, and rs12622919.

^†^ Linear regression adjusted for age, BMI, baseline QTc, and rs12622919.

### Potential of rs12622919 as a Predictor of QTc Prolongation

**Figure 2:**
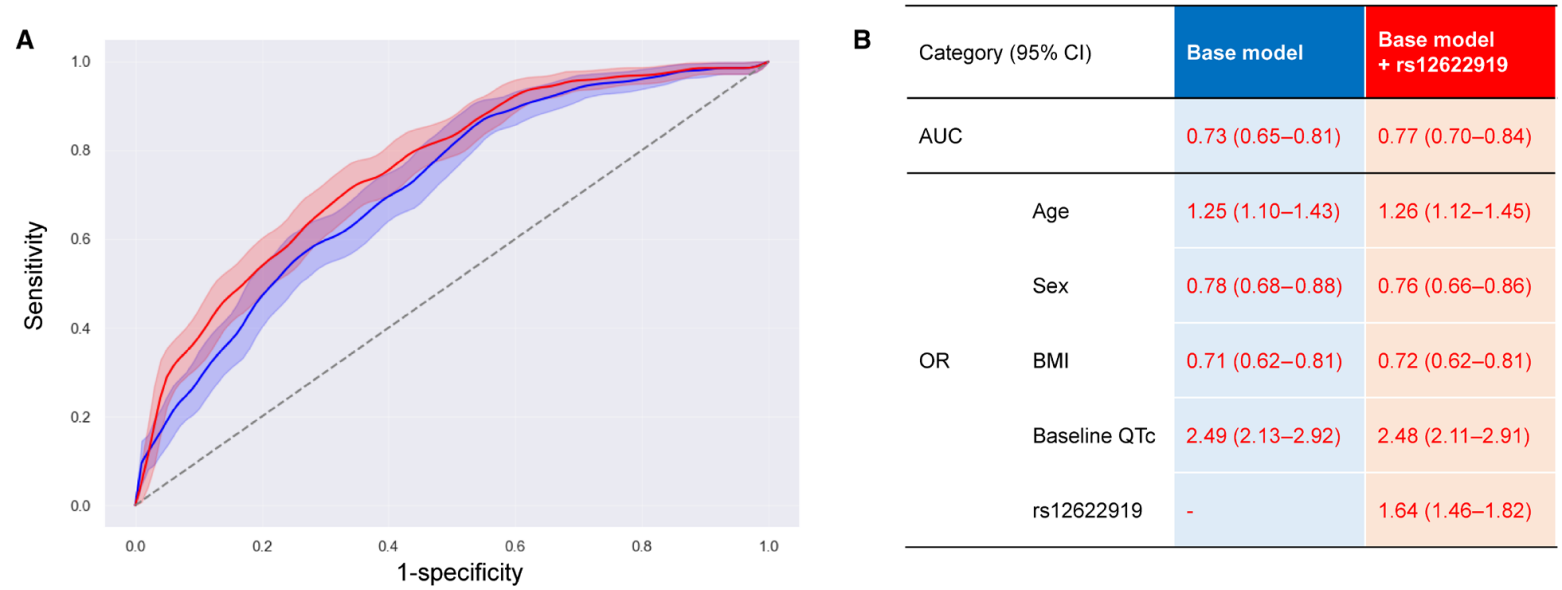
ROC curves comparing the model's performance with and without rs12622919. **A**, Two logistic regression models were constructed: the blue curve indicates the base model, which includes age, sex, BMI, and baseline QTc, and the red curve represents the model, which additionally includes rs12622919 in the base model. **B**, The AUC is significantly higher in the model including rs12622919 than in the model without this variant (Welch's *t* test [*P*<0.001]). AUC indicates area under the curve; QTc, corrected QT interval; and ROC, receiver operating characteristic.

We evaluated the potential of rs12622919 as a predictor of QTc prolongation. Logistic regression models, including age, sex, BMI, baseline QTc, and with or without rs12622919, were constructed using the training set of 390 individuals. Model performance was assessed by calculating the AUC using an independent test set of 131 individuals. The model including rs12622919 achieved an AUC of 0.77 (95% CI, 0.70–0.84), which was significantly higher than that of the model without rs12622919 (0.73 [95% CI, 0.65–0.81]; Welch's *t* test, *P*<0.001; Figure 2). To account for the correlation between receiver operating characteristic curves derived from the same subjects, we additionally compared the AUCs using DeLong's test. Consistent with the initial analysis, the difference between the 2 AUCs remained statistically significant: The model including rs12622919 achieved an AUC of 0.78 (95% CI, 0.74–0.84), whereas the model without rs12622919 achieved an AUC of 0.75 (95% CI, 0.70–0.79; DeLong's test, *P*=0.0078). In the sex‐stratified models, the inclusion of rs12622919 resulted in an AUC of 0.76 (95% CI, 0.66–0.84) in men and 0.80 (95% CI, 0.66–0.93) in women (Table S5).

## Discussions

We performed a GWAS using additive, dominant, and recessive models in patients with QTc prolongation during bepridil therapy and identified the intronic variant of *FSHR* (rs12622919) through the recessive model. The results from a sex‐stratified linear regression analysis and predictive modeling indicated that the rs12622919 variant was significantly associated with QTc prolongation following oral bepridil administration in women, suggesting its potential utility as a biomarker. In contrast, no significant association was observed in men.

The *FSHR* gene encodes the follicle‐stimulating hormone (FSH) receptor, which is a type of gonadotropin secreted by anterior pituitary gonadotropic cells and plays a central role in mammalian reproduction.^29^, ^30^ Its synthesis is primarily regulated by the pulsatile gonadotropin‐releasing hormone secretion. In women, FSH promotes follicle development through actions on granulosa cells, while in men, it regulates Sertoli cell function.^29^

Following menopause, circulating FSH levels rise markedly due to the loss of negative feedback from declining estrogen production. Although the *FSHR* expression in the ovarian tissue decreases, accumulating evidence suggests that *FSHR* may be expressed in extragonadal tissues, including the bone, adipose tissue, and vascular smooth muscles. High FSH levels acting through these receptors have been implicated in increased cardiovascular risk, osteoporosis, and metabolic dysfunction in postmenopausal women.^31^, ^32^ Recent studies have shown that FSH promotes atrial fibrosis in menopausal women with atrial fibrillation.^33^ Therefore, genetic variation in *FSHR* may modulate hormone signaling and indirectly influence cardiovascular physiology.

In patients with torsades de pointes, a hormonal profile characterized by low FSH levels and elevated estradiol has been reported; this is also true in older adults.^34^ These findings suggest that the relative predominance of estradiol in postmenopausal women may promote QTc prolongation via rapid delayed rectifying potassium current (IKr) suppression.^34^, ^35^, ^36^, ^37^ However, ventricular repolarization is maintained by “repolarization reserve” involving slow delayed rectifying potassium current and other K^+^ and Ca^2+^ currents.^35^ Estradiol not only inhibits IKr but also partially compensates by increasing IKs and decreasing the L‐type calcium current; however, this regulation is insufficient to fully offset IKr suppression, resulting in QT prolongation.^38^ In addition, progesterone has been shown to exert protective effects against QT prolongation by enhancing the slow delayed rectifying potassium current and suppressing the L‐type calcium current.^38^ After menopause, progesterone becomes undetectable, whereas low levels of estradiol persist through extraglandular production by adipose tissue. This altered hormonal environment has been proposed to influence repolarization reserve.^37^, ^39^

Xiao et al demonstrated that sustained IKr inhibition leads to a compensatory slow delayed rectifying potassium current increase via posttranscriptional regulation of KvLQT1/mink,^40^ mediated by miR‐133a and miR‐133b.^41^ miR‐133b is also involved in FSH‐dependent estradiol production in ovarian granulosa cells,^42^ suggesting that the FSH–FSHR axis and miRNA‐mediated hormonal regulation may influence ion‐channel expression in the myocardium. These findings indicate that hormone‐dependent regulation of the repolarization reserve may be involved as a factor supporting the association between the FSHR intronic variant (rs12622919) and QT prolongation in women.

Although the functional impact of the intronic *FSHR* variant rs12622919 remains unclear, it may contribute to QTc prolongation by modulating FSH signaling. Further studies are needed to confirm this hypothesis.

Based on the results of the GTEx database, the rs12622919 variant was found to be significantly associated with the reduced expression of *PPP1R21* in the left ventricle. *PPP1R21* is known to enable RNA binding activity and is located in the early endosome.^43^ A previous study has reported that biallelic loss‐of‐function variants in *PPP1R21* cause a neurodevelopmental syndrome associated with endocytic dysfunction.^44^ *PPP1R21* has also been identified as an evolutionarily conserved protein expressed in the developing mouse cortex.^44^, ^45^ It also plays an important role in the endosomal sorting process or endosome maturation pathway^45^ as well as in endosomal trafficking and maturation, which are essential processes for the proper recycling and degradation of membrane proteins, including ion channels.^46^ Impaired *PPP1R21* function may disrupt the recycling of these ion channels, potentially leading to altered action potential duration and QTc prolongation. Although direct evidence linking *PPP1R21* dysfunction to cardiac electrophysiology is currently lacking, our findings raise the possibility that genetic variation affecting *PPP1R21* expression could influence susceptibility to drug‐induced QTc prolongation.

Although the function of rs12622919 remains to be fully elucidated, linear regression analyses and AUC‐based performance evaluations demonstrated its potential utility as a predictive biomarker, even though its effect size was relatively small. Our approach provides a useful framework for investigating QTc prolongation associated with bepridil treatment in the Japanese population. The rs12622919 variant may serve as a predictive marker for QTc prolongation in female patients, and further refinement of this model may lead to practical clinical applications in the future.

### Study Limitations

This study has several limitations.

First, the relatively small cohort size limited the statistical power of our analysis, making it difficult to identify additional SNPs reaching the conventional genome‐wide significance threshold (*P*<5.00×10^−8^). Specifically, the discovery cohort included only 443 patients, and the replication cohort comprised just 78 individuals, representing a major limitation. Although we applied the commonly used GWAS minor allele frequency cutoff of 1%, the limited sample size may have resulted in unstable effect estimates for low‐frequency variants. We also did not adopt the Bonferroni correction for 3 models (additive, dominant, recessive) because of the small sample size. (We set the suggestive significance threshold as *P*=1.00×10^−6^, which would correspond to *P*=0.33×10^−7^ [1.00×10^−6^/3] under Bonferroni correction.) Thus, these findings should be interpreted with caution, as evaluating multiple inheritance patterns without multiple testing correction may increase the likelihood of false‐positive findings.^47^ Although rs12622919 demonstrated robust genome‐wide significance (*P*=8.52×10^−9^), the small sample size should be taken into account.^48^ Moreover, because the primary statistical support derives from the meta‐analysis and the observed association lies near the genome‐wide significance threshold, these findings should be considered preliminary and require confirmation in larger, independent cohorts. The QTc prolongation group was older and had a lower BMI. We cannot exclude the possibility that the small sample size led to imbalances in patient characteristics that influence drug metabolism and disposition, which could bias the associations. In addition, we could not perform a subanalysis distinguishing pre‐ and postmenopausal women because the average age of the cohort was in the mid‐60s. Therefore, the results should be interpreted with caution.^49^, ^50^

Second, QTc prolongation was defined as a QTc of ≥450 ms in men and ≥460 ms in women following bepridil administration; the use of alternative thresholds could have yielded different results.

Third, the baseline QTc was measured at hospital admission for RFCA; however, residual effects of antiarrhythmic drugs may have influenced these measurements. Amiodarone was discontinued 2 weeks before RFCA. Other antiarrhythmic drugs were discontinued 1 week before the procedure; however, their pharmacological effects could still have affected the QTc. Given that the half‐life of amiodarone is ≈53 days, the washout period in our cohort may have been insufficient, and further validation is warranted.^51^ In addition, RFCA for PAF itself may modify the posttreatment QTc.^52^ Prior evidence indicates that QTc prolongation after pulmonary vein isolation is typically transient and tends to improve toward baseline within ≈1 month, suggesting that the influence of ablation alone is likely limited at the time point evaluated in this study.^53^ However, given the observational study design, residual postablation effects and other time‐varying clinical factors cannot be completely excluded.

Fourth, the candidate SNP used in the predictive model was identified using the full data set before model development. Therefore, the internal training–test split cannot fully protect against overfitting, and the predictive performance reported in this study should be interpreted as exploratory rather than confirmatory. Independent external validation in additional cohorts will be required to establish the generalizability and robustness of the prediction model.

Finally, the study cohort consisted exclusively of Japanese individuals, and the generalizability of our findings to other ethnic groups remains uncertain. In addition, we examined eQTLs using the GTEx database; however, its predominantly European ancestry, limited Asian representation, and different age distribution require cautious interpretation.^27^, ^54^

Despite these limitations, our study provides novel insights into the genetic factors contributing to QTc prolongation associated with bepridil administration. The identification of rs12622919 may offer practical value in the future development of biomarkers for risk stratification of bepridil‐prescribed patients. These findings warrant validation through larger, multiethnic, prospective studies.

## Conclusions

The GWAS identified rs12622919 as a genetic variant associated with QTc prolongation during bepridil therapy. Exploratory sex‐stratified analyses suggested a potential association in women; however, no significant genotype‐by‐sex interaction was observed, and this finding should therefore be interpreted with caution. These results indicate that genetic analyses may contribute to risk stratification for QTc prolongation in patients receiving bepridil for PAF. Because the primary statistical evidence is based on meta‐analysis, independent validation in larger cohorts is required to confirm the robustness and clinical utility of this association.

## Sources of Funding

This study was supported by the Japan Society for the Promotion of Science (22K08184), Tokyo, Japan, and partly by the grant from Research Funding for Longevity Sciences from the National Center for Geriatrics and Gerontology (24‐15 to K.O.).

## Disclosures

All the authors declare that they have no competing interests.

## Supporting information

Tables S1–S5
Figure S1

STROBE Checklist
