# Supplementary material for: Genetic Investigation of Corrected QT Interval Sensitivity to Oral Bepridil Hydrochloride Hydrate in Patients With Atrial Fibrillation
Source: J Am Heart Assoc. 2026 Jun 9;15(12):e047046. doi: 10.1161/JAHA.125.047046 (PMC13323570; doi:10.1161/JAHA.125.047046)
Supplement: Supplementary file 1 — Tables S1–S5 Figure S1 [file JAH3-15-e047046-s002.pdf]

SUPPLEMENTAL MATERIAL

Table S1. Demographic data of a replication cohort

Clinical information, mean ± SD or number (%)	QT Prolongation* (n = 17)	non-QT Prolongation (n = 61)	*P* value
age	71.4 ± 6.96	67.1 ± 9.43	0.066
sex (female)	7 (41.12%)	23 (37.70%)	0.79
BMI (kg/m^2)	23.8 ± 3.82	23.9 ± 4.08	0.80
baseline QTc (msec)	432 ± 14.3	416 ± 118	9.91E-4
post-treatment QTc (msec)	465 ± 12.1	433 ± 14.2	1.01E-09
Heart rate (bpm)	63.71±10.35	62.02±10.61	0.39
Blood test			
AST (U/L)	22.59±5.73	23.02±6.9	0.78
ALT (U/L)	20.65±11.81	22.07±10.63	0.14
γ-GTP (IU/L)	39.59±42.61	35.2±21.78	0.45
Creatinine (mg/dl)	0.73±0.17	0.81±0.21	0.17
eGFR (ml/min/1.73m^2)	74.29±14.62	68.89±14.29	0.27
Sodium (mEq/l)	140.29±1.72	139.64±2.32	0.43
Potassium (mEq/l)	4.07±0.27	4.11±0.30	0.59
Chloride (mEq/l)	105.76±2.14	105.03±2.62	0.31
Antiarrhythmic drugs			
Na channel blocker			
Ia	0 (0%)	2 (3.28%)	1
ib	0 (0%)	0 (0%)	
ic	1 (5.88%)	5 (8.20%)	1
Amiodarone	0 (0%)	1 (1.64%)	1
QTc concomitant drugs			
Antibiotics	0 (0%)	0 (0%)	
Antipsychotics	0 (0%)	0 (0%)	
Antidepressants	0 (0%)	1 (1.64%)	1.00
Antihistamines	1 (5.88%)	2 (3.28%)	0.53
Antiepileptic drugs	1 (5.88%)	2 (3.28%)	0.53
Proton pomp inhibitor	5 (29.41%)	37 (60.66%)	0.029
Magnesium oxide	0 (0%)	2 (3.28%)	1.00
Herbal medicine	2 (11.76%)	2 (3.28%)	0.21

Abbreviations: GWAS, genome-wide association study; SD, standard deviation; BMI, body mass index; Aspartate aminotransferase; ALT, Alanine aminotransferase; γ-GTP: γ-glutamyltransferase; eGFR, estimated Glomerular Filtration rate

*QTc prolongation was defined as QTc ≥ 450 ms in males and ≥ 460 ms in females.

Table S2. Primers used for the invader assay

chr	position (hg19)	rsID	forward	reverse	Invader oligo	Invader primer 1	Invader primer 2
2	49306289	rs12622919	ACGACATCACTATGACAGCTGA	CTCCTAAATCCTGGAGCCTGT	CCCCCCATATTATTTTAAACTATATCAATTACAGTGACCACAAAAGTTATACG	CGCGCCGAGGCATATCAAGAAATTAGTCTAAGAAAAAA	ATGACGTGGCAGACTATATCAAGAAATTAGTCTAAGAAAAAA
4	185302070	rs2185830	CTTTTCCCAGGAGCCTCCTC	AGCCACAAACGTTAAGCTTTTGT	GGCCCTGCCCTCATGCCG	CGCGCCGAGGCGGCACAGGTTAGGTTCT	ATGACGTGGCAGACTGGCACAGGTTAGGTTCT
6	130287785	rs957185	CACTACACACTCCTGCCACC	ACCCATTTTCCTCTCAAAGGCA	GGTCAAGCTCTCAGAGTGTTTTCCTTGAAGCCCA	CGCGCCGAGGTTCTCTAGCTTGGCTTAAG	ATGACGTGGCAGACCTCTCTAGCTTGGCTTAAG

Abbreviations: chr, chromosome

Table S3. GTEx-based eQTL estimates for the lead SNP

rsID	chr:position (hg19)	A1	A2	tissue	Ensembl Gene ID	gene symbol	median TPM	beta*	SE	*P* value
rs12622919	2:49306289	C	T	Left ventricle	ENSG00000162869	*PPP1R21*	4.35	-0.10	0.046	0.029

Abbreviations: GTEx, Genotype-Tissue Expression; chr, chromosome; TPM, transcript per million; SE, standard error
*Beta was calculated on the change from A2 to A1.

Table S4. Functional varintas within 1 Mb of rs12622919

gene	SNV	INDEL	total	details (number of variants)
FSHR	16	0	16	nonsynonymous(16)
FOXN2	14	1	15	nonsynonymous(14) frameshift(1)
GTF2A1L	22	0	22	nonsynonymous(20) stopgain(2)
LHCGR	23	0	23	nonsynonymous(23)
NRXN1	6	0	6	nonsynonymous(5) stopgain(1)
PPP1R21	25	1	26	nonsynonymous(24) stopgain(1) frameshift(1)
STON1	1	0	1	nonsynonymous(1)
STON1-GTF2A21L	1	4	32	nonsynonymous(26) stopgain(1) stoploss(1) frameshift(4)
Total	108	6	141	

Abbreviations: SNV, single nucleotide variant; INDEL, insertion and deletion

Table S5. Sex-stratified area under the curve of risk models

sex	category (95% CI)	factor	Base model	Base model + rs12622919
male	AUC		0.73 (0.63 - 0.82)	0.76 (0.66 - 0.84)
	odd ratio	age	1.29 (1.10 - 1.52)	1.28 (1.08 - 1.51)
		BMI	0.88 (0.76 - 1.02)	0.88 (0.75 - 1.01)
		baseline QTc	2.50 (2.13 - 2.97)	2.45 (2.07 - 2.92)
		rs12622919	-	1.53 (1.31 - 1.78)
female	AUC		0.76 (0.61 - 0.88)	0.80 (0.66 - 0.93)
	odd ratio	age	1.14 (0.94 - 1.38)	1.20 (1.00 - 1.45)
		BMI	0.48 (0.37 - 0.60)	0.50 (0.38 - 0.62)
		baseline QTc	2.21 (1.68 - 3.20)	2.30 (1.72 - 3.44)
		rs12622919	-	1.75 (1.41 - 2.19)

Abbreviations: AUC, area under the curve; CI, confidence interval

Figure S1. Regional plots of candidate SNPs with suggestive associations.

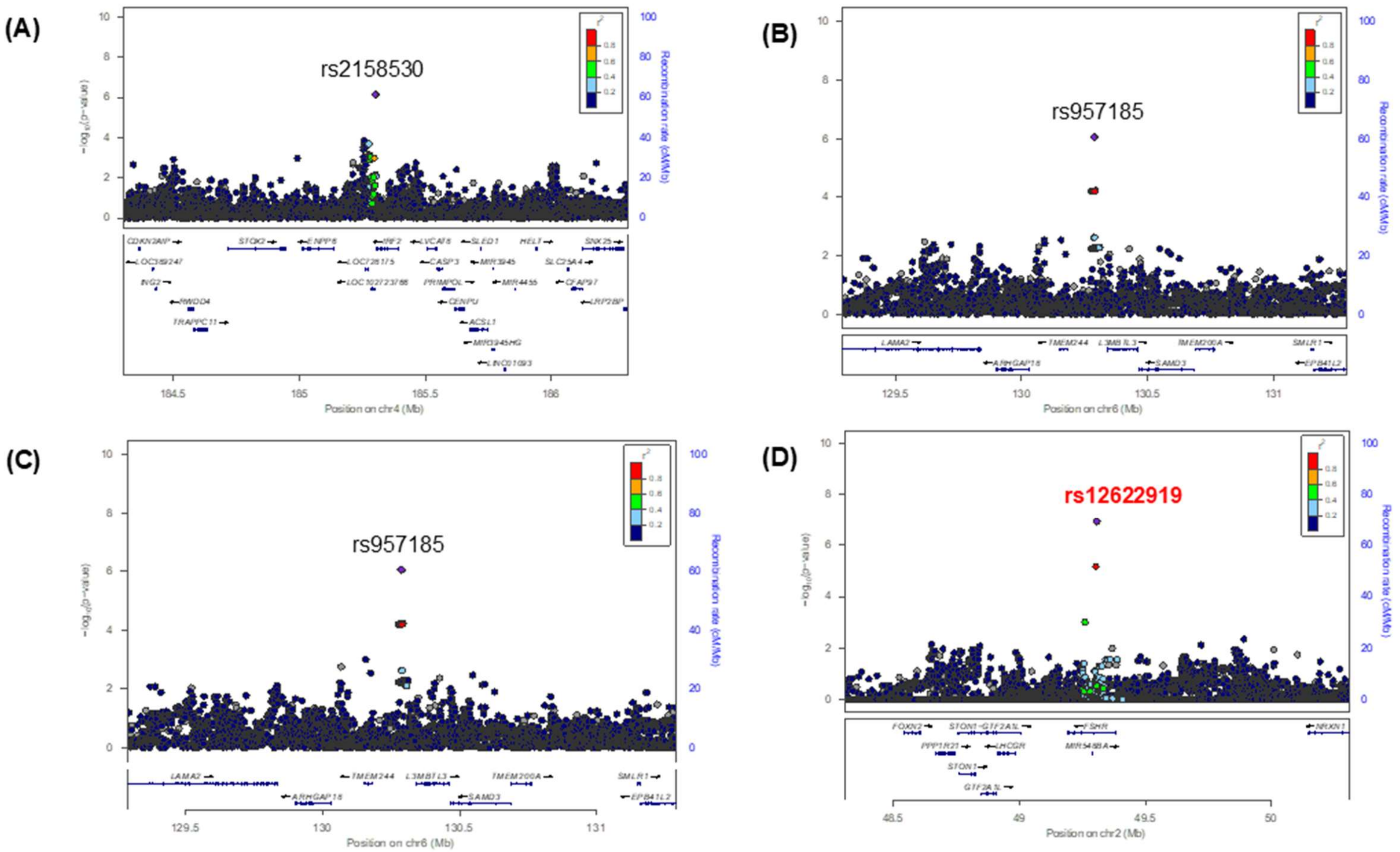

(A) and **(B)** show loci surrounding rs12622919 analyzed under the additive genetic model; **(C)** shows the loci analyzed under the additive model; **(D)** shows loci analyzed under the recessive model.
